# Evaluation of quality and utility of YouTube vitreoretinal surgical videos

**DOI:** 10.1186/s40942-022-00360-w

**Published:** 2022-02-02

**Authors:** Luiz Filipe Adami Lucatto, Juliana Moura Bastos Prazeres, Ricardo Luz Leitão Guerra, Rafael Arantes, Gabriel Castilho Sandoval Barbosa, Emmerson Badaró, Luiz H. Lima, Eduardo Rodrigues

**Affiliations:** 1 Ophthalmology Department, Federal University of São Paulo (UNIFESP/EPM), R. Botucatu—822, São Paulo, SP 04023-900 Brazil; 2 Ophthalmology Department, Obras Sociais Irmã Dulce, Salvador, Bahia Brazil; 3 Ophthalmology Department, Suel Abujamra Institute, São Paulo, SP Brazil; 4 Ophthalmology Department, Saint Louis University Eye Institute (SLUEI), St. Louis, MO USA

**Keywords:** Surgical learning, YouTube videos, E-learning, Vitreoretinal surgery

## Abstract

**Background:**

We evaluated the utility and quality of surgical videos posted on the main retinal YouTube channels by surgeons at different career stages and assessed how well the steps of the vitrectomy videos conformed to the parameters in the Casey Eye Institute Vitrectomy Indices Tool for Skills assessment (CEIVITS) scale.

**Methods:**

Forty-five videos were included from nine retinal YouTube channels posted from 2011 to 2021. For each surgeon, 10 videos were randomized and the utility, quality, and educational content were assessed. For each video, the surgeons also assessed how the validated CEIVITS items were presented in the videos. The surgeons were divided based on years of experience: fellows (0–3 years), young surgeons (4–10 years), and senior surgeons (more than 10 years).

**Results:**

The video image quality was rated as good in 63.52% of evaluations, moderate in 30.37%, and poor in 6.11%. The quality assessment of the videos among the groups did not differ. The fellows rated the use of the videos as educational tools higher (3.99) than the young (3.87) and senior surgeons (3.47) (p  < 0.0002, Kruskal–Wallis test); 34.76% of the fellows reported learning something new from the videos compared with 19.17% of the senior surgeons (p  < 0.05). The CEIVITS scale item that was seen more frequently was related to core vitrectomies (72.29%) and the least represented was about checking infusion lines (80.17%).

**Conclusions:**

Vitreoretinal surgical videos are useful educational tools during all stages of surgeons’ careers, and the evaluation of the quality of the images did not differ significantly among the groups, however, surgeons with expertise shorter than 10 years report significantly greater use of videos than experienced surgeons. Videos posted to the public domain on different social media, most often YouTube, are widespread and unregulated for providing complimentary surgical education. Retinal societies should formulate guidelines and improve the educational value of the surgical videos posted on the Internet.

*Trial Registration* The Federal University of São Paulo institution’s Research Ethics Committee reviewed and approved this study protocol (Approval Number, 4.726.589).

**Supplementary Information:**

The online version contains supplementary material available at 10.1186/s40942-022-00360-w.

## Background

Traditionally, surgical training has been undertaken with the mentor and mentee participating in face-to-face training during a procedure. Constant technologic evolution in the medical field, however, results in trainees dealing with surgical techniques of greater complexity and the necessity to acquire a greater volume of knowledge during their training. In addition, opportunities to observe and learn surgical procedures can be hampered by geographic barriers and political institutions that emphasize operating room efficiency [1]. The learning curve associated with vitreoretinal surgery can be long and requires the development of key surgical skills. Medical professionals today have access to several tools, and the use of online resources has become part of the educational process [2]. Surgical videos are an example in which the combination of figures, captions, and diagrams can be added to facilitate learning.

Studies have demonstrated the benefits of multimedia in learning by converting cognitive input into long-term memory [2, 3]. A wide range of free online retinal surgical videos is available on different social media sites, which provides access to information in a fast, practical, and inexpensive way, and eliminates the limitations imposed by geographic and time barriers [4, 5]. Studies in several medical areas have reported that YouTube currently is the main platform used by surgeons to prepare for surgical procedures [1, 2]. Surgical videos on YouTube are tools that can be useful and appropriate to complement surgical learning, assuming that the content has an educational purpose and the video has good quality and detailed explanations. Both experienced surgeons and trainees can benefit from watching surgical video content, either to review a rarely performed procedure, improve technical details, or discover different solutions that other colleagues have performed. However, the quality and utility of surgical videos on YouTube should be questioned since there is no adequate control, and the content can be posted by anyone, without a review and quality-control process. In addition, the perception of the utility of the videos and critical analysis about the presence of inappropriate content may vary depending on the experience of the surgeon viewing the video.

No currently available publications discuss how the vitreoretinal surgical videos available on YouTube are evaluated for their utility and quality. The current study assessed how retinal surgeons at different career stages assess the utility and quality of retinal surgical videos currently available on retinal YouTube channels.

## Methods

### Study design

This was a cross-sectional survey study in which an online survey was sent to vitreoretinal specialists and fellows via the Google Forms website. A comprehensive search was carried out on YouTube (https://www.youtube.com) on January 18, 2021, using the search term retina surgery. The YouTube channel filter was selected for the search. The videos on each channel were evaluated and selected according to the following criteria: videos posted between January 2011 and 2021; length, shorter than 10 min; subject, surgeries to treat vitreoretinal diseases; videos created by professionals for professionals; presentation in English; and a minimum of 1000 views on the platform. Educational and institutional videos designed to provide patient information or marketing were excluded. A total of 1054 videos from 12 YouTube channels were listed, and from these, the top five videos on each channel based on the number of views were selected. Of these 12 channels, three were excluded because they did not have a total of five videos that satisfied the inclusion criteria. Of the 45 videos selected, 10 were randomized for retinal surgeons at different career stages to evaluate.

### Evaluation of quality and utility as an educational tool

For each selected video, basic characteristics such as the number of days online, duration, number of views, topic covered, presence of subtitles, presence of narration, number of comments, and number of likes and dislikes were analyzed. The descriptions of the channels included the number of views, followers, and number of videos posted. The overall image quality of the videos was rated as poor, moderate, or good. The use of the videos as an educational tool for vitreoretinal surgery was classified using the Likert 5-point scale (1 useless to 5 very useful). For each video, the examiners answered the following questions “Have you learned something new from this video?” and “Did you see something inappropriate or controversial in the video’s educational content?” To assess surgical performance, examiners applied the items from the Casey Eye Institute Vitrectomy Indices Tool for skills Assessment (CEIVITS) to each video, which was validated as a tool to assess basic vitrectomy maneuvers [6]. The CEIVITS is comprised of 10 items including positioning and incisional techniques for sclerotomies; preparation, positioning and checking the infusion line; surgical microscope focus and navigation; technique for removing the core vitreous; membranectomy technique; fluid-air exchange, and sclerotomy sutures. Each domain is accessed on the 5-point Likert scale (1 indicating worst to 5 best). For each item, the examiners scored 0 points if a step was not visible in the video, 1 if it was partially visible, and 2 if it was clearly visible. If the surgical step was unrelated to the procedure shown in the video, the examiner marked it as “does not apply”. Data extracted and parameters evaluated are shown in Table 1. The Channel and Videos characteristics are detailed in Additional file 1: Table S1.

**Table 1:** Data extracted for each retinal educational channel on YouTube and the parameters of each video

Participant
Age
Country
Time of career (years)
Quality assessment
Image quality (poor, moderate, good)
Utility assessment
Likert scale (1 indicates not useful and 5 indicates very useful)
Educational content
“Have you learned something new from this video?” (yes/type or no)
“Did you see something inappropriate or controversy in the video’s educational content?” (yes/type or no)
CEIVTS scale
For each item of the scale
“Surgical step was not shown on video”
“Surgical step was partially shown on video”
“Surgical step was clearly shown on video”
“Surgical step does not apply to this procedure”

### Participants

Three retinal surgeons and investigators produced the initial form, 15 retinal surgeons who volunteered to participate as a pre-test group tested and modified the form. During the pre-test study, the adequacy of each question was assessed, questions poorly formulated and options for answers were identified and corrected. The form was further shortened to reduce fatigue and improve the overall style according to guidelines for conducting and reporting survey research [7, 8]. In the second phase of the study, 75 retinal surgeons with different levels of experience were invited to participate in the survey through e-mail or online messaging applications that contained a link to an online form generated using Google forms. Retinal surgeons and fellows were invited from lists of retinal medical societies and contacted individually. The project was described on the first page, and informed consent was obtained from all participants. Upon agreeing to participate, the evaluator was directed to a page containing three questions about demographic data (age, country, and length of career as a retinal specialist). The evaluator then watched the videos and answered three multiple-choice questions (required), one on a linear scale (required), and two descriptive (optional), and classified each of the 10 videos in a table according to the parameters of the CEIVITS scale [6].

All responses were anonymous and stored in a password-protected account on the server that generated the survey. Two authors (LFAL and JMBP) analyzed the results. The Federal University of São Paulo institution’s Research Ethics Committee reviewed and approved this study protocol. The data collected during the survey remained confidential.

### Statistical analysis

Data analysis was performed using Stata/SE 12.0 (StataCorp LLC, College Station, Texas, USA). Descriptive statistics are presented as frequencies (n) and percentages (%) for categorical variables and means or medians [standard deviation (SD) and range] for continuous and ordinal variables. The internal consistency among the examiners was assessed through Cohen’s Kappa coefficient. The value of 0.41 was considered as moderate. Spearman’s rho was calculated to assess the degree of correlation among quality, utility, and numbers of likes and views. Pearson’s chi-square test was used to verify differences between groups regarding learning and the controversial or inappropriate video content. The Kruskal–Wallis test was performed to assess the differences in quality and use of the videos among the three groups. p values less than 0.05 indicated statistical significance.

## Results

### Channel selection process and video characteristics

**Fig. 1:**
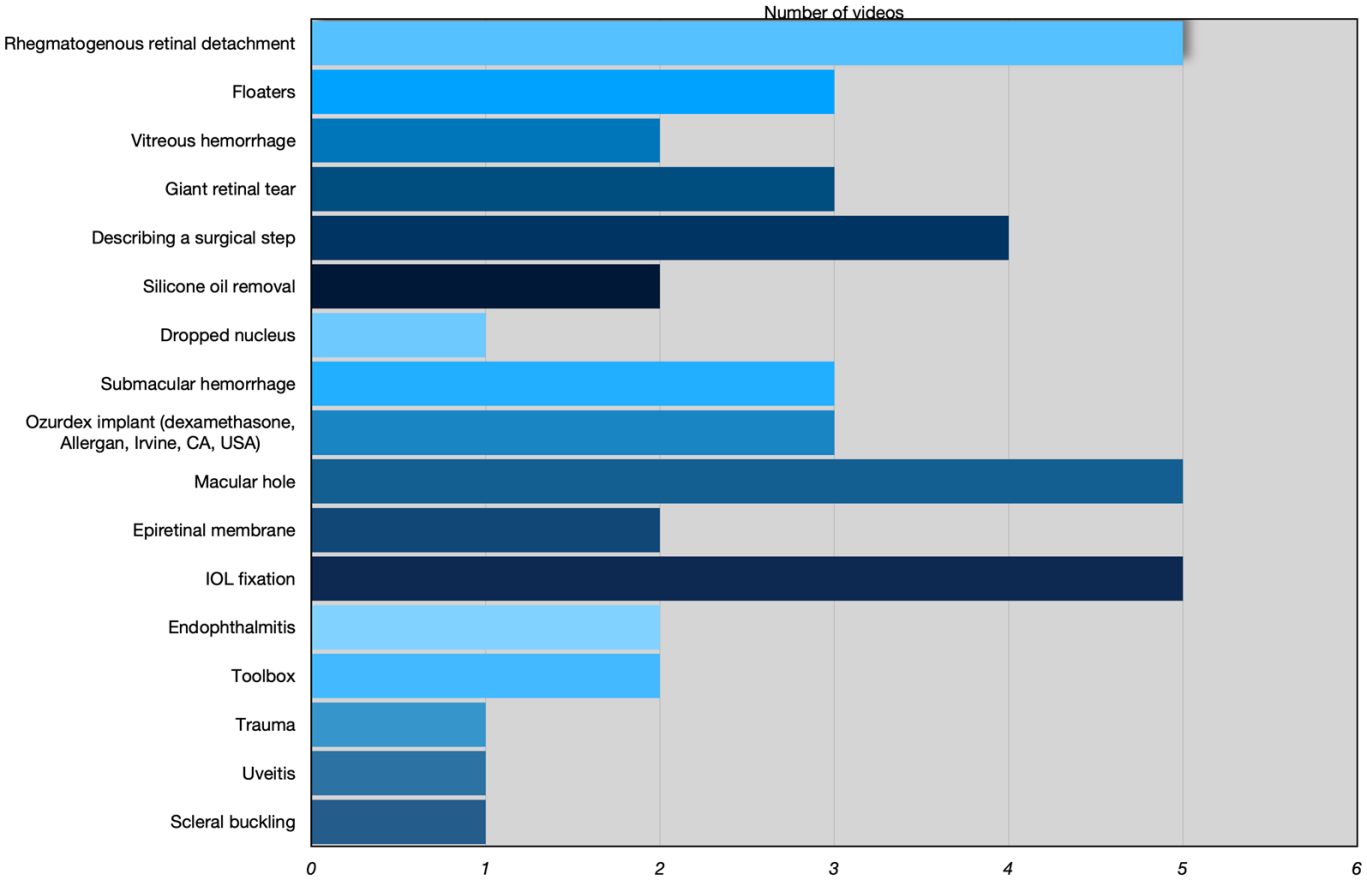
List of all topics and their frequency in the study sample

The search retrieved videos from 29 retina channels on YouTube. Each channel was analyzed individually, and the eligibility of its videos was checked. The characteristics of the YouTube channels in the vitreoretinal surgeries and of each of the 45 selected videos are shown in Additional file 1: Table S1. Overall, three channels (33.33%) were from Europe, two (22.22%) from Asia, two (22.22%) from North America, one (11.11%) from South America, and one (11.11%) from Oceania. On average, these retinal channels had 145,566.67 views (range, 54,500–295,000) and 1937.44 followers (range, 339–4490). Selected videos were available online for a mean of 55.23 months (range, 4.93–113.3). The average duration of the videos was 4.37 min (standard deviation, 2.30; range, 1.17–9.95 min). The videos received an average of 38.04 comments (range, 0–505), with more likes (average, 104.22; range, 10–894) than dislikes (average 6.96; range 0–81). Written commentaries were present in 93% of the cases and narration only in 36%. The topics most covered in the sample videos were rhegmatogenous retinal detachments, macular holes, and intraocular lens fixation (5 videos each). The complete list of topics covered and the respective frequency of each is shown in Fig. 1.

### Participants

Of the 75 retinal surgeons invited to participate, 54 (39 men, 15 women) agreed to participate in the study (72%). The average age of the participants was 35.28 years (range, 27–51), and the average career duration was 6.81 years (range, 0–25 years). For data analysis, surgeons were classified by career length as fellows (0–3 years), young surgeons (4–10 years), and senior surgeons (more than 10 years). Thus, the sample of 54 participants included 38.89% of fellows, 38.89% of young surgeons, and 22.22% of senior surgeons. Retinal surgeons from 12 countries participated in the study, with 78% from Latin America, 9% from North America, 6% from Central America, 4% from Asia, and 4% from Europe. Table 2 shows the characteristics of the participants.

**Table 2:** Demographic data from the 54 participants and their levels of surgical experience

Sex	
Male	39
Female	15
Total	54
Age	
Mean (range)	35.28 years (27–51 years)
Country	
Brazil	39
Dominican Republic	3
Mexico	3
France	1
Colombia	1
Argentina	1
Japan	1
Canada	1
Iraq	1
Chile	1
Turkey	1
USA	1
Length of career	
Mean (range)	6.81 years (0–25 years)
Fellows (0–3 years)	21
Young surgeon (4–10 years)	21
Senior surgeon (> 10 year)	12

### Quality and utility assessment

The image quality of each video was rated as poor (0), moderate (1), and good (2). The overall average quality of the videos analyzed in the study was 1.57 (SD, 0.61). The video quality was rated as good by 63.52%, moderate by 30.37%, and 6.11% as poor. The quality assessment of the videos among the groups (Table 3) did not differ significantly (p  = 0.2502). The utility of the surgical videos as an educational tool rated on the Likert scale from 1 to 5 was an average of 3.83 (SD, 1.16); 65% of the videos received a score of 4 or 5, and 18.7% received a score of 1 or 2. The fellows awarded an average utility score of 3.99, the young surgeons 3.87, and the senior surgeons 3.47. The utility assessments among the groups differed significantly (p  = 0.0002) (Table 3). Among the evaluated parameters, a correlation was seen between the utility and quality of the videos (p  = 0.0011) and between the number of views and number of likes (p  < 0.0001). No correlations were seen between image quality and utility and the number of video views on YouTube.

**Table 3:** Quality and utility assessment among the groups

Quality	
Mean ± SD	1.57 ± 0.61
Fellows (0–3 years)	1.59
Young surgeons (4–10 years)	1.62
Senior surgeons (> 10 years)	1.48
	p = 0.2502
Utility	
Mean ± SD	3.83 ± 1.16
Fellows (0–3 years)	3.99
Young surgeons (4–10 years)	3.87
Senior surgeons (> 10 years)	3.47
	p = 0.0002

### Educational content analysis

In 29.6% of all evaluations, surgeons responded that they learned something new by watching the video; 34.76% (p  < 0.05) of the fellows reported learning something new. Despite the significant difference, the senior surgeons responded that they learned something new in 19.17% of the evaluations. Regarding the presence of controversial or inappropriate educational content in the video, 33.33% of the senior surgeons reported inappropriate content versus 21.90% of the fellows (p  = 0.069). The general average found for this question was 26.85%.

### CEIVITS scale assessment

**Fig. 2:**
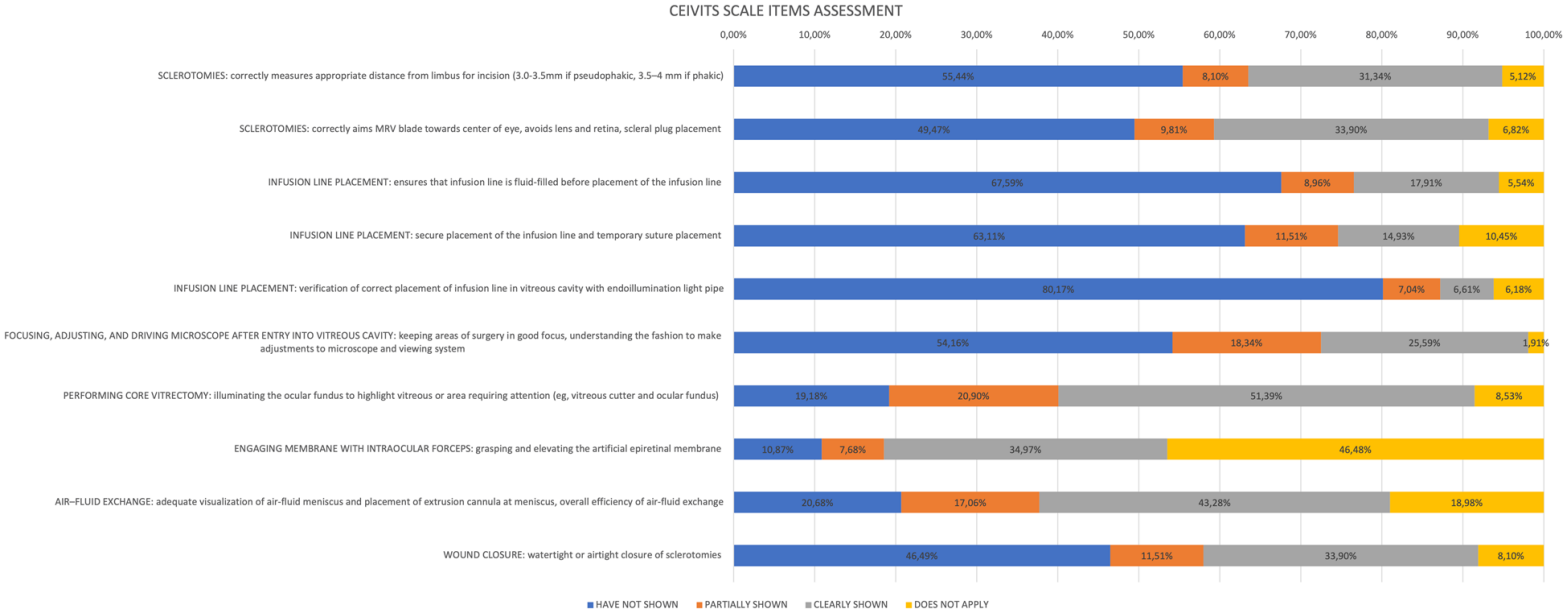
Frequencies of each item in the Casey Eye Institute Vitrectomy Indices Tool for Skills Assessment Scale in the 39 vitrectomy videos

Regarding items on the CEIVITS scale assessment, 39 videos related to vitrectomy surgeries were analyzed. Six videos were removed from this subanalysis because the steps on the scale did not apply to the type of surgery covered by the video. The CEIVITS scale items that were partially or clearly showed more frequently were related to core vitrectomy (72.29%) and fluid-air exchange (60.34%). The items less frequently showed were about checking infusion line (absent in 80.17%) and infusion line fluid-filling (absent in 67.59%). The complete list of CEIVITS scale items is shown in Fig. 2.

## Discussion

This study presents a detailed assessment of the quality and utility of vitreoretinal surgical videos in the public domain and available on YouTube based on the perspective of surgeons at different career stages. We evaluated the top five videos in number of views from nine different channels of vitreoretinal surgery, selected according to the inclusion criteria on January 18, 2021. These videos have been viewed on the platform by more than 1 million people worldwide and have been available online for an average of 4.53 years. Considering the growing number of vitreoretinal surgical videos posted on YouTube and other platforms and the trend toward increasing use of social media among surgeons, it is important for the scientific community to verify the educational value of these new e-learning tools [9–12]. The importance increases when considering that currently there are no control tools that evaluate the posted content, and there are no guidelines proposed by medical retinal societies to guide how to properly prepare a surgical video in this area. However, since the evolution of technology reduces limitations by geographic locations and access to qualified mentors, surgeons-in-training can watch surgical videos on the Internet, and this allows greater contact with different surgical techniques, facilitating the search for additional information in scientific articles and discussions with more experienced colleagues.

We assessed if the image quality and utility of retinal surgical videos were graded differently based on career durations. As expected, the evaluation of the quality of the images did not differ significantly among the groups. Previous studies in different medical fields have reported the generally poor image quality of the YouTube videos [13–15]. Unlike these studies, the image quality of the retinal surgical videos was not a limitation in the current sample. Only 6% of the videos were considered poor; 60% of the videos were rated as good and 33% as moderate. The greater access to new technologies that acquire high-quality images in surgical cameras, especially in retinal surgeries with availability of recordings captured using 3-dimensional visualization systems such as NGENUITY^®^ (Alcon, Fort Worth, TX, USA), tend to improve the content of the images shown in conference discussions and posted on the Internet.

Regarding use of videos as an educational tool, the current analysis showed that more inexperienced surgeons (fellows or those with careers shorter than 10 years), reported significantly greater use of videos than experienced surgeons (with careers longer than 10 years). Even with the difference observed between the senior surgeons and the other two groups, the average utility score in this group was 3.47 on a scale of 1–5. The general average of the study sample was 3.83. Similarly, the analysis of the educational content found that the fellows learned something new from the videos 34.76% of the time (p  < 0.05), and the more experienced surgeons reported learning something new 19.17% of the time. This result indicates that videos can be useful even for experienced surgeons. The retinal specialty has a wide range of techniques and different surgical indications, and, therefore, the surgical videos available online can be important sources of complementary knowledge. The topics most frequently addressed in the current sample were rhegmatogenous retinal detachments, macular holes, and intraocular lens implantation in the absence of adequate capsular support. The complexity and range of techniques to treat retinal detachments and the recent publication of different techniques for treating macular holes and intraocular lens fixation justify the surgeons’ greater interest in these topics [16–18]. However, a rate of 26.85% for controversial and/or inappropriate content was found in the videos. Contrary to expectations, no significant difference was seen among the groups, and even the fellows had similar rates in the critical evaluation of the videos compared to the surgeons in the more experienced groups.

The image quality and the utility of the videos were parameters that were significantly correlated. However, the number of views on the platform was not correlated with any of these parameters. The videos in the current sample had subtitle content 93% of the time but narration only 36% of the time. Guidelines from other medical fields recommend the use of diagrams, photos, tables, and audio/written content in English to provide additional educational content [14].

International multispecialty committees recently have published a statement consensus on how to present laparoscopic surgical videos for educational purposes (LAP-VEGaS) [13, 14]. The retinal specialty does not have such a tool to show the conformity of the surgical steps of vitrectomy with surgical videos. In the current study, we used the CEIVITS scale, which is published and used as a tool to access basic maneuvers in vitrectomy and aid surgeons in training [6]. The scale highlights 10 important items in the surgical steps of vitrectomies and grades the surgeon’s performance on a scale of 1–5. We applied the items in the scale to the current study, so that the examiner selected how the surgical steps were presented in the videos. The items most demonstrated in the videos were core vitrectomy and fluid-air exchange. Important steps that must be remembered for beginning surgeons related to technique, positioning, and checking the infusion line are steps usually removed from the videos.

A limitation of the survey is the subjectivity in the classification of videos regarding quality (poor, moderate, or good) and utility (5-point Likert scale), as well as answering the two descriptive optional questions. Furthermore, although we included participants from 12 different countries, the vast majority reside in Brazil (72%), and there may be regional variations in training experience that this research could not discern.

These results emphasize the need for a standardized system on how to present retinal surgical videos on the Internet. As already carried out by some medical subspecialties, international committees can meet to publish a consensus to guide creation of high-quality educational videos. It is difficult to adopt control and validation measures for surgical videos on YouTube and other social media since there is no such entity responsible for reviewing the material. In this way, therefore, any surgeon can make the content available on the Internet without any filters.

To our knowledge, no study has been published that assessed the quality and usefulness of retinal surgical videos. The current results show that videos can be useful, high-quality tools for complementary surgical learning among retina specialists at any career stage. However, important measures to standardize the editing and the content of these videos should be studied and published to reduce the chances of including educationally inappropriate materials.

## Conclusions

Vitreoretinal surgical videos represent a useful educational tool for surgeons at all career stages. Videos available in the public domain, most often on YouTube, are widespread and unregulated for complementary surgical education. Retinal societies should formulate guidelines and improve the educational value of the posted surgical videos on the Internet.

## Supplementary Information

**Additional file 1: Table S1.** Characteristics of the nine YouTube channels in vitreoretinal surgery (ordered by number of subscribers, January 18, 2021). The characteristics of the five most viewed videos of each channel are shown.

## Data Availability

The authors are responsible for the data in the manuscript and assure full availability of the study material.

## Abbreviations

CEIVITS: Casey Eye Institute Vitrectomy Indices Tool for skills Assessment
SD: Standard deviation
