# Supplementary material for: Evaluation of quality and utility of YouTube vitreoretinal surgical videos
Source: Int J Retina Vitreous. 2022 Feb 2;8:9. doi: 10.1186/s40942-022-00360-w (PMC8812238; doi:10.1186/s40942-022-00360-w)
Supplement: Supplementary file 1 — Additional file 1: Table S1. Characteristics of the nine YouTube channels in vitreoretinal surgery (ordered by number of subscribers, January 18, 2021). The characteristics of the five most viewed videos of each channel are shown. [file 40942_2022_360_MOESM1_ESM.docx]

|  | **SUPPLEMENTARY TABLE 1**  **Characteristics of Nine YouTube Channels in Vitreoretinal Surgery (Ordered by Number of Subscribers, January 18, 2021). Characteristics of the Five Most Viewed Videos of Each Channel Are Shown** | | | | | | | | | | | | | |
| --- | --- | --- | --- | --- | --- | --- | --- | --- | --- | --- | --- | --- | --- | --- |
|  | **Channel** | **Channel views** | **Subscribers** | **Channel videos** | **Video** | **Title and Link** | **Number of views** | **Number of days online** | **Length (min)** | **Comments** | **Likes** | **Dislikes** | **Subtitles** | **Narration** |
|  | **Randall Wong - USA** | 136,000 | 4490 | 65 |  |  |  |  |  |  |  |  |  |  |
|  |  |  |  |  | 1 | Scleral Buckle and Vitrectomy for Retinal Detachment https://www.youtube.com/watch?v=CJy8qjNojAc&t=6s | 296,488 | 3290 | 7.03 | 505 | 894 | 81 | Yes | Yes |
|  |  |  |  |  | 2 | Vitrectomy for Vitreous Floaters (FOV) https://www.youtube.com/watch?v=JJ-BK9cKJ4w&t=35s&has_verified=1 | 175,169 | 3161 | 2.83 | 432 | 407 | 44 | Yes | Yes |
|  |  |  |  |  | 3 | 25 g. Vitrectomy for Vitreous Hemorrhage (Graphic!) https://www.youtube.com/watch?v=lF82FOGtLRs | 150,306 | 3143 | 3.05 | 196 | 325 | 33 | Yes | Yes |
|  |  |  |  |  | 4 | Repair of Giant Retinal Tear https://www.youtube.com/watch?v=8yTue0CNTwM | 56,797 | 2631 | 4.9 | 60 | 235 | 12 | Yes | Yes |
|  |  |  |  |  | 5 | Inducing a PVD (Posterior Vitreous Detachment) During FOV (Floater Only Vitrectomy) https://www.youtube.com/watch?v=hcOYZMGdiZk | 46,327 | 2535 | 3.83 | 86 | 227 | 9 | Yes | Yes |
|  | **Ulrich Spandau - Sweden** | 67,000 | 3500 | 439 |  |  |  |  |  |  |  |  |  |  |
|  |  |  |  |  | 6 | 13.10 Cerclage removal https://www.youtube.com/watch?v=G8iOSgUCF5M | 39,439 | 1691 | 3.63 | 1 | 27 | 10 | Yes | No |
|  |  |  |  |  | 7 | 6.3 Silicone oil removal https://www.youtube.com/watch?v=xkPQlGttII8&t=118s | 21,328 | 1102 | 6.43 | 28 | 114 | 14 | Yes | No |
|  |  |  |  |  | 8 | 20.2 Surgery of a dropped nucleus with Infinity and 3 trocars https://www.youtube.com/watch?v=M-A6J7PmDQs | 6,920 | 1086 | 8.3 | 20 | 63 | 4 | Yes | No |
|  |  |  |  |  | 9 | 16.3 Submacular Actilyse (rtPA) for hemorrhagic AMD https://www.youtube.com/watch?v=6YrVuRXK5W0 | 6,296 | 1975 | 5.77 | 18 | 45 | 2 | Yes | No |
|  |  |  |  |  | 10 | 13.9 How to place an encircling band (cerclage) https://www.youtube.com/watch?v=xHQowcjqQpw | 4,973 | 1480 | 6.63 | 8 | 46 | 0 | Yes | No |
|  | **Mumbai Eye Retina Clinic - India** | 148,000 | 2180 | 37 |  |  |  |  |  |  |  |  |  |  |
|  |  |  |  |  | 11 | Ozurdex (Allergan) Intravitreal Implant \| Retina Injection \| Dr Madhusudan Davda https://www.youtube.com/watch?v=O3GBnzn0SeA | 30,954 | 1243 | 1.73 | 3 | 66 | 5 | Yes | No |
|  |  |  |  |  | 12 | Macular Hole Surgery \| How to peel ILM? \| Dr Madhusudan Davda, Mumbai Eye Retina Clinic https://www.youtube.com/watch?v=cRInp0gHfSA&t=39s | 6,869 | 1285 | 2.93 | 3 | 27 | 2 | Yes | No |
|  |  |  |  |  | 13 | Macular Hole Surgery \| MIVS Surgery \| Dr Madhusudan Davda \| Mumbai Eye Retina Clinic https://www.youtube.com/watch?v=Pc-JxZkdrq0 | 4,797 | 2027 | 3.83 | 3 | 14 | 0 | Yes | No |
|  |  |  |  |  | 14 | ERM peeling \| Retina Surgery \| Dr Madhusudan Davda \| Mumbai Eye Retina Clinic https://www.youtube.com/watch?v=h-7dsirH3mA | 2,664 | 1278 | 2.78 | 2 | 11 | 2 | Yes | No |
|  |  |  |  |  | 15 | Myopic Macular Hole with Retinal Detachment - ILM peeling in extreme situations https://www.youtube.com/watch?v=Iy1cITy_AbI | 2,020 | 863 | 2.93 | 2 | 10 | 1 | Yes | No |
|  | **Diego Ruiz Casas / VR Surgery - Spain** | 295,000 | 1920 | 168 |  |  |  |  |  |  |  |  |  |  |
|  |  |  |  |  | 16 | Retinal detachment vitrectomy Tips / Consejos en vitrectomía para DR https://www.youtube.com/watch?v=7iW7FagxRPM | 15,773 | 1009 | 6.83 | 45 | 123 | 3 | Yes | No |
|  |  |  |  |  | 17 | How to implant an Iris-Claw IOL retropupillary https://www.youtube.com/watch?v=sTJokpELwt8 | 9,011 | 1252 | 4.63 | 14 | 53 | 11 | Yes | No |
|  |  |  |  |  | 18 | Advanced Endophthalmitis https://www.youtube.com/watch?v=hyMKs9s4cVs | 6,981 | 1258 | 6.4 | 44 | 61 | 0 | Yes | No |
|  |  |  |  |  | 19 | Ozurdex https://www.youtube.com/watch?v=TktjJjb_CQY | 5,553 | 1793 | 1.5 | 0 | 13 | 2 | No | No |
|  |  |  |  |  | 20 | Non-contact BIOM Focus and Navigation Technique https://www.youtube.com/watch?v=pQNHnfCVsRg&t=29s | 5,456 | 869 | 9.38 | 19 | 94 | 0 | Yes | No |
|  | **Retina Tips - Brazil** | 72,100 | 1730 | 100 |  |  |  |  |  |  |  |  |  |  |
|  |  |  |  |  | 21 | Intraocular lens scleral fixation: Pearls on Yamane Technique https://www.youtube.com/watch?v=duMZyJlqCWc | 8,833 | 424 | 4.15 | 14 | 125 | 2 | Yes | Yes |
|  |  |  |  |  | 22 | Surgical steps - Primary pars plana vitrectomy for rhegmatogenous retinal detachment https://www.youtube.com/watch?v=0ixy2uoTUrM&t=34s | 4,013 | 148 | 4.0 | 16 | 62 | 2 | Yes | Yes |
|  |  |  |  |  | 23 | Subretinal injection of tPA in submacular hemorrhage https://www.youtube.com/watch?v=N9W9vE69A58&t=180s | 2,418 | 432 | 4.32 | 1 | 61 | 0 | Yes | Yes |
|  |  |  |  |  | 24 | Improving visualization using BIOM system in vitreoretinal surgery https://www.youtube.com/watch?v=eIrD1KLpKpY&t=79s | 2,076 | 454 | 2.43 | 9 | 60 | 2 | Yes | Yes |
|  |  |  |  |  | 25 | Pinch and peel technique (ILM peeling) - Author: Filipe Lucatto MD https://www.youtube.com/watch?v=wdR8ocULSPE | 1,723 | 524 | 1.17 | 0 | 20 | 0 | No | Yes |
|  | **Dr. Manish Nagpal - India** | 242,000 | 1730 | 70 |  |  |  |  |  |  |  |  |  |  |
|  |  |  |  |  | 26 | Vitrectomy for Silicon Oil Removal \| SOR \| Retinal Detachment https://www.youtube.com/watch?v=hTnk8vd8I2Q&t=73s&has_verified=1 | 57,051 | 3397 | 7.82 | 33 | 165 | 16 | Yes | Yes |
|  |  |  |  |  | 27 | Vitrectomy for Retinal Detachment \| PVR \| ! A step by step approach \| Dr Manish Nagpal https://www.youtube.com/watch?v=M5FEoQxgep4&t=240s | 27,158 | 3398 | 7.87 | 12 | 109 | 10 | Yes | Yes |
|  |  |  |  |  | 28 | Vitrectomy for various indications \| PVD creation \| Dr Manish Nagpal https://www.youtube.com/watch?v=ALIUm035pUg&t=20s | 23,287 | 3059 | 9.95 | 32 | 130 | 3 | Yes | Yes |
|  |  |  |  |  | 29 | Vitrectomy for Giant Retinal Tear \| GRT \| Retinal Detachment \| Dr Manish Nagpal https://www.youtube.com/watch?v=u9JwtI2J4Xg | 22,222 | 3399 | 7.73 | 14 | 106 | 4 | Yes | Yes |
|  |  |  |  |  | 30 | Vitrectomy for Retinal Detachment \| PVR \| Retinectomy \| Dr Manish Nagpal https://www.youtube.com/watch?v=lP-Ogb8ISyU | 16,594 | 2615 | 6.82 | 8 | 107 | 4 | Yes | Yes |
|  | **Dr. Simon Chen - Australia** | 221,000 | 1140 | 20 |  |  |  |  |  |  |  |  |  |  |
|  |  |  |  |  | 31 | Floater Only Vitrectomy (FOV) for MASSIVE vitreous eye floaters - Dr Simon Chen HD 1080p https://www.youtube.com/watch?v=lHaG3Cktq5k | 63,556 | 1423 | 1.87 | 12 | 355 | 14 | Yes | No |
|  |  |  |  |  | 32 | Dislocated IOL: Yamane technique, Vitrectomy & Intrascleral Haptic Fixation ISHF - Dr Simon Chen HD https://www.youtube.com/watch?v=kPIVGy0V2ZQ | 30,269 | 1558 | 5.3 | 22 | 171 | 10 | Yes | No |
|  |  |  |  |  | 33 | Giant retinal tear repair - vitrectomy surgery - Dr Simon Chen 1080p HD https://www.youtube.com/watch?v=nxRzQro55jA&t=46s | 7,352 | 1978 | 2.63 | 10 | 58 | 1 | Yes | No |
|  |  |  |  |  | 34 | Dislocated IOL/capsular bag: Scleral Fixation Sutures / Hoffman pockets / Vitrectomy - Dr Simon Chen https://www.youtube.com/watch?v=H9zsRuYRQ2U | 6,533 | 1558 | 3.55 | 3 | 43 | 0 | Yes | No |
|  |  |  |  |  | 35 | Subretinal air & vitrectomy surgery for submacular haemorrhage due to AMD - Dr Simon Chen 1080p HD https://www.youtube.com/watch?v=81BgPeLuNAU | 2,687 | 1576 | 2.07 | 9 | 18 | 0 | Yes | No |
|  | **Dr. Med. Nikolaos Tsiampalis - Ophthalmic Surgeon - Germany** | 74,500 | 408 | 38 |  |  |  |  |  |  |  |  |  |  |
|  |  |  |  |  | 36 | intraocular foreign body removal https://www.youtube.com/watch?v=40m2D_lFqfg | 10,991 | 1826 | 2.58 | 4 | 58 | 3 | Yes | No |
|  |  |  |  |  | 37 | Ozurdex Injection https://www.youtube.com/watch?v=dnI8qlHdI4Y | 5,840 | 1941 | 1,.48 | 3 | 13 | 0 | Yes | No |
|  |  |  |  |  | 38 | vitreous washout https://www.youtube.com/watch?v=kzJcBo_MMNg&t=42s | 4,250 | 1884 | 1.77 | 1 | 20 | 0 | Yes | No |
|  |  |  |  |  | 39 | vitrectomy - uveitis https://www.youtube.com/watch?v=u_qfQHxMEvo | 4,088 | 1892 | 3.07 | 8 | 22 | 2 | Yes | No |
|  |  |  |  |  | 40 | 23G vitrectomy for endophthalmitis https://www.youtube.com/watch?v=LED_u6GiBAI | 4,005 | 2250 | 5.83 | 2 | 13 | 1 | No | No |
|  | **Dr. Aureliano Moreno RC Tijuana - Mexico** | 54,500 | 339 | 81 |  |  |  |  |  |  |  |  |  |  |
|  |  |  |  |  | 41 | Modified Inverted ILM Flap Technique For Large Macular Holes https://www.youtube.com/watch?v=-w1W20W_SlA | 3,209 | 1095 | 2.32 | 3 | 41 | 0 | Yes | No |
|  |  |  |  |  | 42 | Modified Inverted ILM Flap Technique for Macular Hole After Vitrectomy for Retinal Detachment https://www.youtube.com/watch?v=nsPYI9anc98 | 2,266 | 577 | 2.12 | 2 | 18 | 1 | Yes | Yes |
|  |  |  |  |  | 43 | Yamane Trans-Scleral Fixation Technique For Secondary Intraocular Lens Implantation \| Tijuana, Mex. https://www.youtube.com/watch?v=UUF7fD4FQoQ | 1,979 | 427 | 3.77 | 0 | 27 | 1 | Yes | No |
|  |  |  |  |  | 44 | Diabetic Macular Edema Treatment \| Retina Center Tijuana https://www.youtube.com/watch?v=cp6Dq0uvpeI | 1,728 | 974 | 3.1 | 2 | 16 | 1 | Yes | No |
|  |  |  |  |  | 45 | Epiretinal Membrane Peeling. Vitrectomy in Macular Edema Associated with Thick Epiretinal Membrane. https://www.youtube.com/watch?v=Q-Ah1Cwbto0&t=35s | 1,594 | 777 | 3.62 | 3 | 17 | 1 | Yes | No |
